# Supplementary material for: Changes in temperature alter the potential outcomes of virus host shifts
Source: PLoS Pathog. 2018 Oct 19;14(10):e1007185. doi: 10.1371/journal.ppat.1007185 (PMC6209381; doi:10.1371/journal.ppat.1007185)
Supplement: S1 Text — (DOCX) [file ppat.1007185.s001.docx]

**
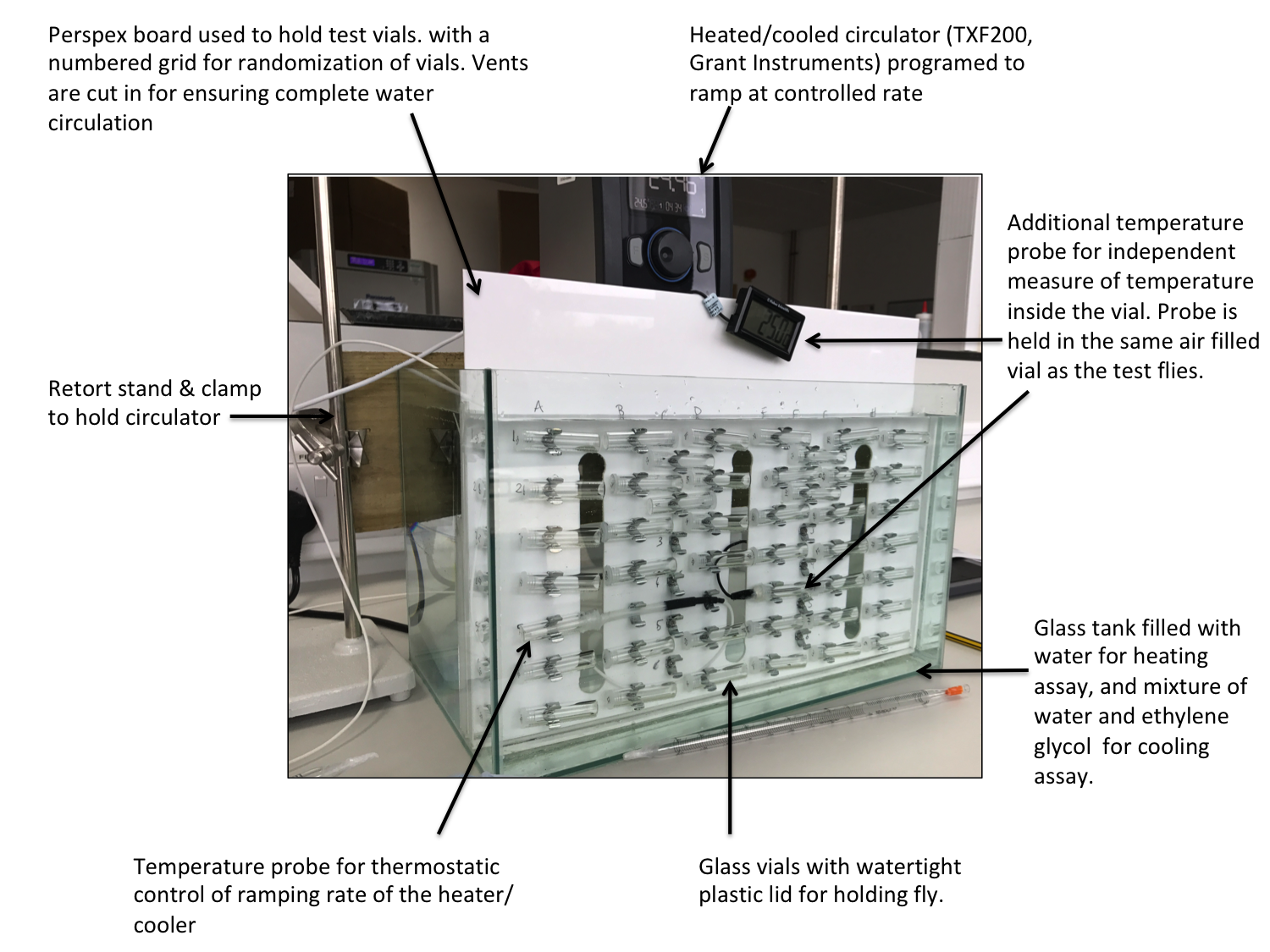
S1 Text Fig A. Critical Thermal Maximum and Minimum Assay Equipment set up**

**Critical Thermal Maximum and Minimum Assay Equipment list:**

- Water tank
- Heater/cooler pump (TXF200, Grant Instruments, Cambridgeshire, UK)
- 4 ml glass vials (ST5012 Ampulla)
- Terry clips
- Perspex board
- Temperature probe (Fisherbrand Traceable Digital Thermometer #S90862)
- Parafilm – for making waterproof seal for temperature probe held in tubes
- Water
- Ethylene Glycol
- Stand and clamps to secure equipment

**S1 Text Methods: Measurement Correction Model Methods:**

Due to the error level in the Basal Metabolic Rate (BMR) data we were unable to fit an efficient model that could account for this noise. It is therefore difficult to fit standard models that account for this error when including BMR as a fixed effect. We therefore fitted a model that allows for error in fixed effects. This allowed us to use the raw BMR data in which the effect of respiratory chamber number was controlled for, and species by temperature effects were fitted as random. Viral load was then essentially regressed against the species BMR effects by ordering traits (BMR followed by viral load) within temperatures and fitting a first order interdependence structure to the covariance matrix. This results in a single regression coefficient of viral load on BMR that is constant over temperatures, and is analogous to fitting BMR as a main effect in a model of viral load had species-mean BMRs been measured with 100% accuracy. Ideally the model would have allowed the between species variance in viral loads and BMR to be different across different temperatures and would have allowed the between species effects to be correlated between temperatures. Unfortunately, MCMCglmm does not have this flexibility, and so we ran two models; one where the variances differ but the correlation is zero (model A), and one where the variances are identical but the correlations are one (model B). We also reran both these analyses using species effects that were correlated due to phylogeny (model E and F respectively). Treating species mean BMR by viral load as independent across temperatures resulted in a negative regression slope (-34.2 (95% CI: -66.0, -7.3) and -48.0 (95% CI: -76.3, -16.1) with phylogeny) as did treating species mean BMR by viral load as identical across temperatures (-18.5 (95% CI: -51.3, 13.0) and -9.8 (95% CI: -62.5, 42.6) with phylogeny).

The low repeatability of BMR resulted in wide credible intervals for the relationship between viral load and BMR making it hard to draw firm conclusions. However, in all models there was evidence of substantial between-species variance in viral load after conditioning on BMR suggesting that BMR, even if it had of been measured accurately, could not explain the observed between-species patterns.

**S1 Text Table A**: Full list of species used in the experiment and their rearing food for stock populations. All cornmeal and Proprionic medium have dried yeast sprinkled onto the surface of the food, other food types do not unless stated below. The recipes for the food medium reared on are as follows: banana recipe, cornmeal recipe, proprionic recipe and malt recipe.

| **Species** | **Food** |
| --- | --- |
| *D.affinis* | Malt |
| *D.americana* | Malt |
| *D.ananassae* | Cornmeal |
| *D.arizonae* | Banana |
| *D.bifasciata* | Malt |
| *D.buzzatii* | Malt |
| *D.erecta* | Malt + yeast |
| *D.euronotus* | Cornmeal |
| *D.flavomontana* | Malt + yeast |
| *D.hydei* | Cornmeal |
| *D.immigrans* | Malt + yeast |
| *D.lacicola* | Malt |
| *D.littoralis* | Banana |
| *D.lummei* | Malt + yeast |
| *D.mauritiana* | Proprionic |
| *D.melanogaster* | Cornmeal |
| *D.micromelanica* | Cornmeal |
| *D.mojavensis* | Banana |
| *D.montana* | Malt + yeast |
| *D.nasuta* | Cornmeal |
| *D.novamexicana* | Banana |
| *D.obscura* | Proprionic |
| *D.paramelanica* | Cornmeal |
| *D.persimilis* | Malt |
| *D.prosaltans* | Proprionic |
| *D.pseudoobscura* | Malt |
| *D.saltans* | Cornmeal |
| *D.santomea* | Cornmeal |
| *D.sechellia* | Proprionic |
| *D.simulans* | Cornmeal |
| *D.sturtevanti* | Cornmeal |
| *D.subobscura* | Cornmeal |
| *D.sucinea* | Cornmeal |
| *D.takahashii* | Cornmeal |
| *D.teissieri* | Cornmeal |
| *D.virilis* | Proprionic |
| *D.yakuba* | Cornmeal |
| *H.duncani* | Proprionic |
| *S. rufifrons* | Cornmeal |
| *S.lebanonensis* | Proprionic |
| *S.pattersoni* | Banana |
| *Z. davidi* | Banana |
| *Z. inermis* | Banana |
| *Z. taronus* | Banana |
| *Z. tuberculatus* | Banana |

**S1 Text Table B:** qRT-PCR primers. Drosophila *RpL32* primers were designed to match the homologous sequence in each species and crossed an intron-exon boundary so will only amplify mRNA (Longdon *et al.* 2011).

| qRT-PCR primer name and location/species | Sequence |
| --- | --- |
| *RpL32* qRT-PCR F (*D. melanogaster*) | TGCTAAGCTGTCGCACAAATGG |
| *RpL32* qRT-PCR R (*D. melanogaster*) | TGCGCTTGTTCGATCCGTAAC |

1. **Longdon B, Hadfield JD, Webster CL, Obbard DJ, Jiggins FM. Host Phylogeny Determines Viral Persistence and Replication in Novel Hosts. Schneider DS, editor. PLoS Pathog. (2011) ;7: e1002260. doi:10.1371/journal.ppat.1002260**

**S1 Text Fig B (a)** Viral load for 6 *Drosophilidae* species at 27°C, on day 0, day 1 and day 2 post infection. Points are the means of three replicates (± S.E.) of 10 male flies, that were housed, as per the main experiment, at 27°C for 5 days before being experimentally infected with DCV. Viral loads are measured relative to the house keeping genes (*Rpl32*) for each time-point*.* **(b)** Change in viral load for the same 6 *Drosophilidae* species across the three temperatures (Low= 17°C, Medium=22°C and High=27°C). Data already presented in main text (Fig. 1) and shown here for comparison with the time course in (a).


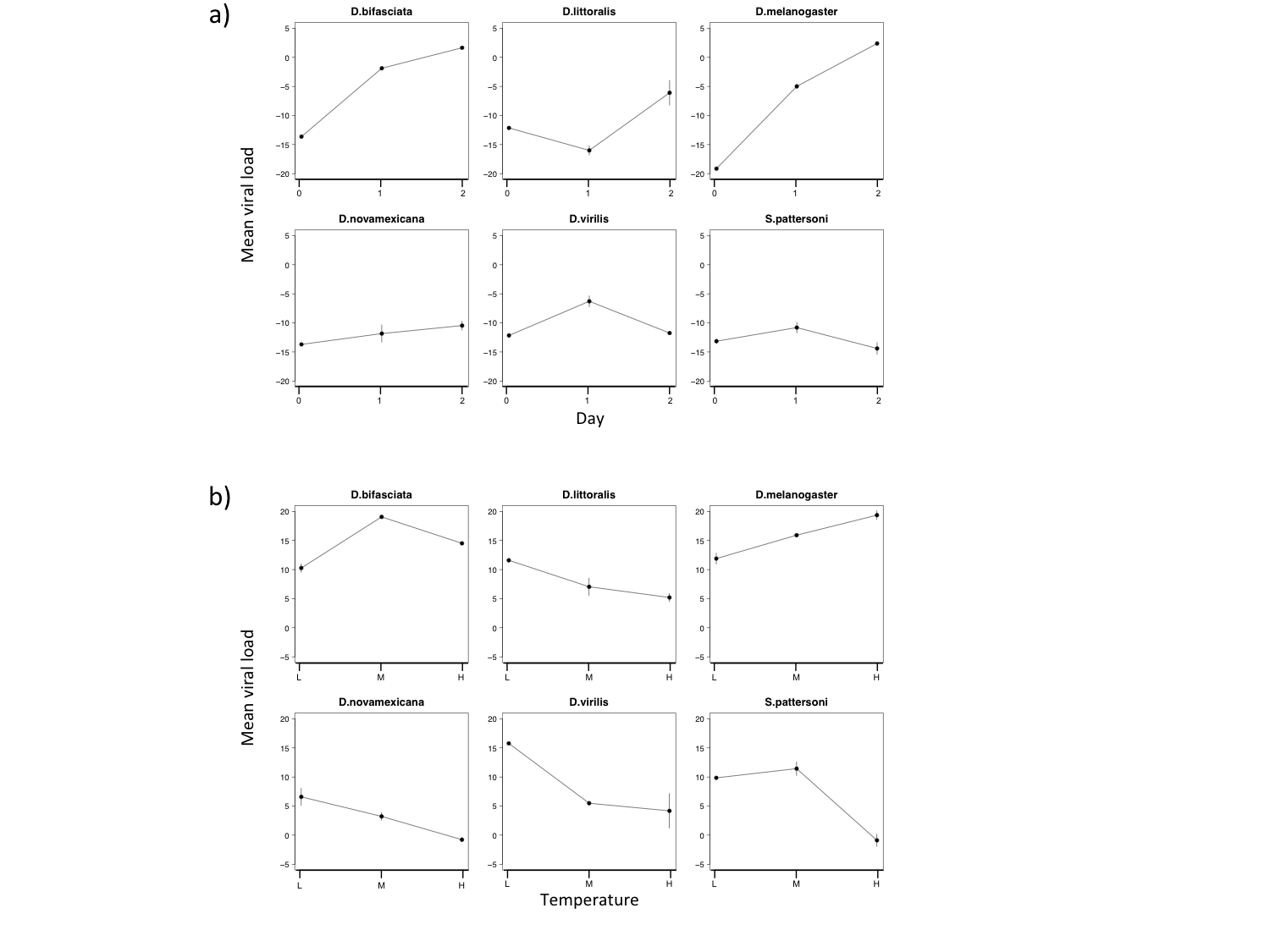

**S1 Text Fig C: Ancestral state reconstructions of CT_min_ and CT_max_ for experimental flies**; for CT_min_ assays temperatures were gradually lowered and CT_max_ they were gradually raised until flies were paralysed, and the temperature was recorded to the nearest (0.1°C). Colours represent the knock down temperature with red representing the highest temperature and green the lowest temperatures that caused total paralysis.

**S1 Text Fig D: Change in Basal Metabolic Rate (BMR) for 44 *Drosophilidae* species across three temperatures**. The y-axis is the rate of CO_2_ production (VCO_2_) as a proxy for metabolic rate the x- axis is the temperature treatment (Low= 17°C, Medium=22°C and High=27°C). *D. pseudobscura* was excluded as not enough individuals could be obtained for replicate measures. Individual points are for each replicate and the red line is the mean rate for each species. Panel ordering is that of tips on the phylogeny.


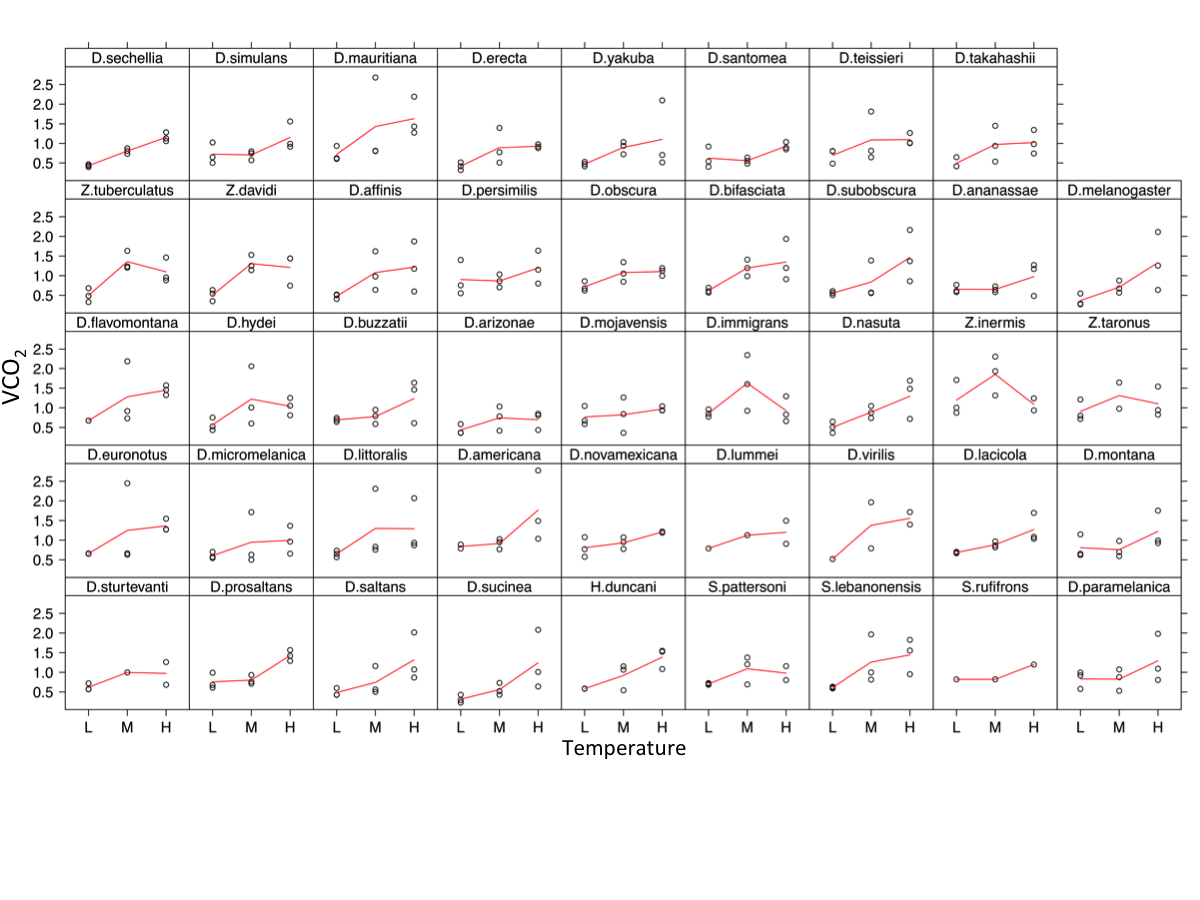


**S1 Text Fig E: Ancestral state reconstructions of BMR across the three experimental temperatures;** with red representing the highest level and green the lowest level VCO_2,_ (rate of CO_2_ production) as a proxy for metabolic rate at that temperature.


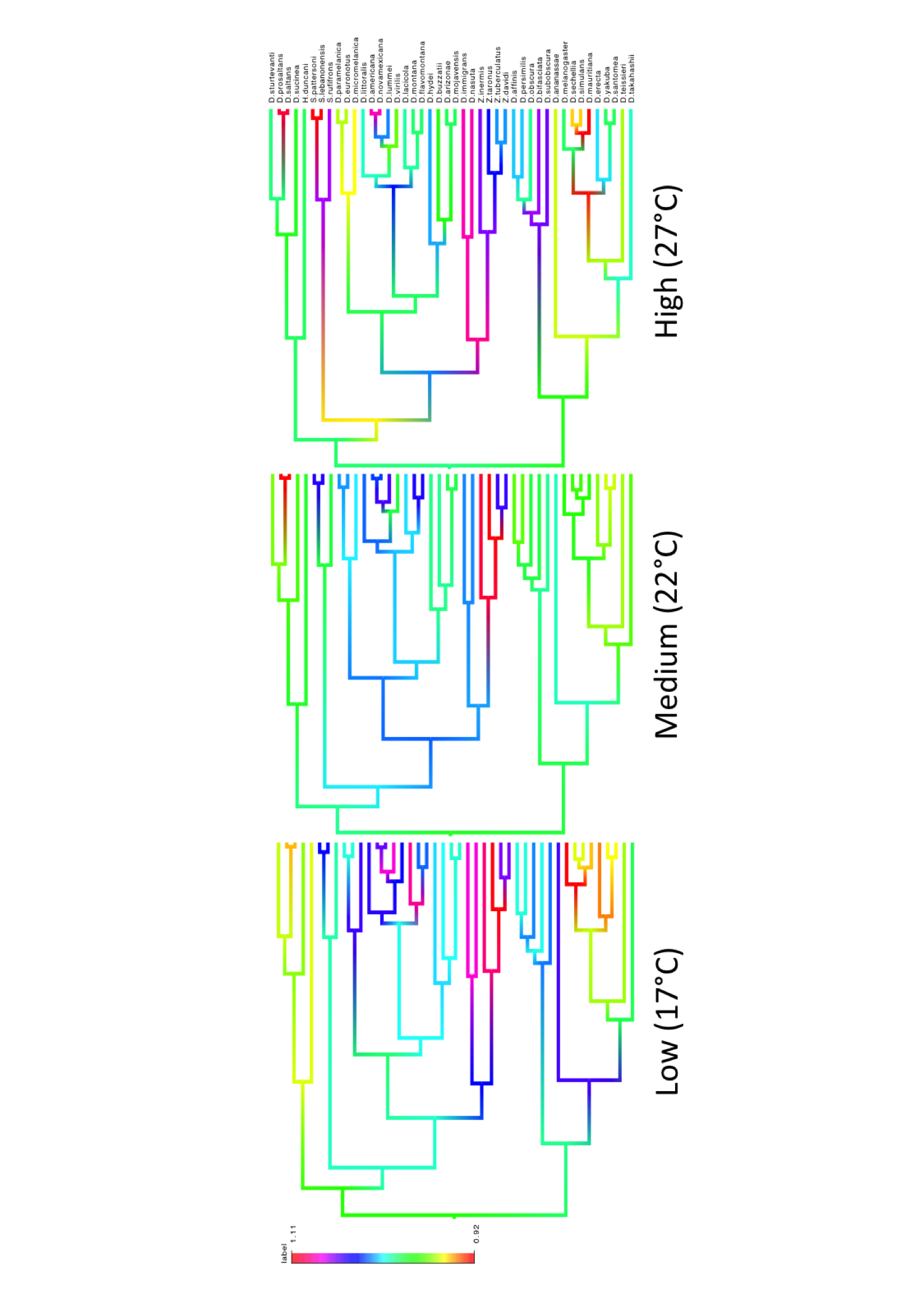


**S1 Text: Full project dataset:** <https://figshare.com/account/home#/projects/30692>

- Genbank accession numbers of sequences used to infer the host phylogeny (downloaded sequences are in coloured black) sequences generated during this project are in orange: https://figshare.com/account/projects/30692/articles/6653192
- Full dataset for main analysis: https://figshare.com/account/projects/30692/articles/6037979
- Ctmax/Ctmin data: https://figshare.com/account/projects/30692/articles/6034466
- Metabolic rate data: https://figshare.com/account/projects/30692/articles/6040970
- R code: https://figshare.com/account/projects/30692/articles/6177191
